# Pustular Psoriasis and Acute Generalized Exanthematous Pustulosis

**DOI:** 10.3390/medicina57101004

**Published:** 2021-09-23

**Authors:** Morgan Sussman, Anthony Napodano, Simo Huang, Abhirup Are, Sylvia Hsu, Kiran Motaparthi

**Affiliations:** 1 Department of Dermatology, Lewis Katz School of Medicine at Temple University, Philadelphia, PA 19140, USA; morgan.sussman@temple.edu (M.S.); simo.huang@tuhs.temple.edu (S.H.); sylvia.hsu@tuhs.temple.edu (S.H.); 2 Department of Dermatology, University of Florida College of Medicine, Gainesville, FL 32610, USA; anapodano@ufl.edu; 3 College of Medicine, University of Florida, Gainesville, FL 32606, USA; acare2019@hotmail.com

**Keywords:** pustular psoriasis, acute generalized exanthematous pustulosis, severe cutaneous adverse reaction, chronic inflammatory disorder

## Abstract

The similarity between pustular psoriasis (PP) and acute generalized exanthematous pustulosis (AGEP) poses problems in the diagnosis and treatment of these two conditions. Significant clinical and histopathologic overlap exists between PP and AGEP. PP is an inflammatory disorder that has numerous clinical subtypes, but all with sterile pustules composed of neutrophils. AGEP is a severe cutaneous adverse reaction that is also characterized by non-follicular sterile pustules. Clinical features that suggest a diagnosis of PP over AGEP include a history of psoriasis and the presence of scaling plaques. Histologically, eosinophilic spongiosis, vacuolar interface dermatitis, and dermal eosinophilia favor a diagnosis of AGEP over PP. Importantly, PP and AGEP vary in clinical course and treatment. PP treatment involves topical steroids, oral retinoids, and systemic immunosuppressants. Newer therapies targeting IL-36, IL-23, IL-1, and PDE-4 have been investigated. The removal of the offending agent is a crucial part of the treatment of AGEP.

## 1. Introduction and Epidemiology

Pustular psoriasis (PP) and acute generalized exanthematous pustulosis (AGEP) share similar clinical and histopathologic findings. Historically, AGEP was first described as a variant of PP [1]. A subset of patients within a case series of 104 patients with PP demonstrated an acute pustular eruption that spontaneously resolved without recurrence [1]. These patients lacked a prior history of psoriasis, and the etiology of the eruption was purported to be secondary to medication or infection. Characterization of AGEP as a distinctive entity from PP did not occur until many years later [2]. Importantly, the differentiation of AGEP from PP remains challenging in many instances but is worthwhile due to differences in management and prognosis. A summary of the features of PP and AGEP can be found in Table 1.

PP is a chronic inflammatory disorder comprised of several distinct subtypes, including generalized pustular psoriasis (GPP), impetigo herpetiformis (IH), palmoplantar pustular psoriasis (PPPP), acrodermatitis continua of Hallopeau (ACH), and annular pustular psoriasis (APP). A uniting factor among each subtype is the presence of sterile, non-infectious pustules characterized by an intraepidermal neutrophilic infiltration on histopathology [3]. Further, there is an overlap in genetic factors and treatment responses [3]. PP can exist in conjunction with psoriasis vulgaris (PV), the most prevalent variant of psoriasis, or entirely on its own [4,5]. The co-occurrence of the two conditions has necessitated the inclusion of PP within the psoriasis spectrum, although this has historically been debated. Of note, PV is most often seen in patients with the PPPP subtype [6]. The prevalence of PV in patients with PPPP is 10–25 times higher compared to the general population [6]. Psoriasis vulgaris comprises about 80% of cases of psoriasis, while PP makes up the remaining 20% of cases [3]. GPP is most frequently seen in association with PV, with about half of GPP patients presenting with both conditions [5]. PPPP is the most common variant of PP [3,4], with a prevalence of 0.050 to 0.12% [7]. The overall rarity and heterogeneity of PP presents challenges in uncovering its genetic basis, correlations between genotype and phenotype, and pathophysiology [3,5].

AGEP is a severe cutaneous adverse reaction (SCAR) that displays significant overlapping clinical and histopathologic features with PP. The incidence of AGEP is estimated to be 1–5 cases per million patients per year with a mean age of 56 years [17,18]. Women are affected more commonly than men at a 3:0.8 ratio [18]. In greater than 90% of cases, the inciting trigger can be traced to drugs, usually certain classes of antibiotics, including penicillins and macrolides. While classic AGEP can resemble PP by morphology, cases of atypical AGEP that resemble toxic epidermal necrolysis (TEN) and drug-induced hypersensitivity syndrome (DIHS) have also been described [19,26]. These TEN-like and DIHS-like AGEP cases share overlapping clinical features, indicating the polymorphic presentation of AGEP.

## 2. Pathophysiology

Disruption of the interleukin-36 pathway plays a critical role in PP pathophysiology [9,10]. Further, the innate immune system, environmental factors, and genetic susceptibility also contribute to the disease [7]. Approximately one-third of patients with PP contain monogenic mutations [3]. It is established that mutations in three genes involved in the innate immune system, *IL36RN*, *AP1S3*, and *CARD14*, play a role in the pathogenesis of PP [3,4,5]. However, these target genes are mutated in only a small fraction of PP cases, with variations among each PP subtype [3,5]. This highlights the need for further exploration of disease pathogenesis. A 2019 study by Twelves et al. compared the major genetic differences between GPP, PPPP, and ACH [5]. Most commonly, homozygous and compound heterozygous loss-of-function mutations were reported in all three conditions [4,5]. Of the three genes, *IL36RN* is the most frequent genetic aberration found in patients with PP, with 23.7% of patients with GPP harboring this mutation [5]. *IL36N* encodes a negative regulator for the IL-36 receptor, referred to as the IL36 receptor antagonist [4,5]. The IL36 receptor antagonist inhibits the effects of several pro-inflammatory cytokines, including IL-1F6, IL-1F8, and IL-1F9 [4]. As a result, mutations in the regulator lead to uncontrolled activation of the pro-inflammatory pathways mediated by the cytokines [4], whose downstream effects include activation of NFkB and release of inflammatory markers [3,5]. In all three subtypes, the *IL36RN* mutation was associated with an earlier age of onset [5]. Screening for this mutation may guide future treatment, which is now beginning to target the IL36RN pathway [4,5]. Additionally, *IL36RN* mutations have been associated with a more severe clinical course in patients with GPP [27]. Patients present at an earlier age with an increased risk of systemic inflammation [27]. Of note, APP shares a similar pathogenesis, likely involving the IL-36 signaling pathway [16].

In PPPP, eccrine sweat glands are involved in the inflammatory process [7]. An increased number of Langerhans cells has been documented surrounding the eccrine sweat glands, leading to an infiltration of inflammatory cells that release inflammatory markers responsible for destroying the sweat glands and driving pustule formation. Specifically, increased levels of IL-8, IL-17, and IL-36γ have been detected in biopsies [7]. Overexpression of these cytokines is likely induced by antimicrobial peptides, which suggest an antigen-driven process by Langerhans cells. The release of IL-8 leads to neutrophil chemoattraction and pustule formation on the palmoplantar surfaces.

There are two congenital syndromes, deficiency of IL-36 receptor antagonist (DITRA) and deficiency of IL-1 receptor antagonist (DIRA), that involve similar pathways. As seen in PP, both conditions involve loss-of-function mutations in genes regulating the innate immune system [15]. DITRA involves an autosomal recessive mutation in the *IL36RN* gene, resulting in the uncontrolled inflammatory cascade present in PP [15]. DIRA is characterized by an autosomal recessive mutation in the IL-1 receptor antagonist gene (*IL-1RA*), leading to a partial or complete absence of the receptor antagonist and subsequent uncontrolled activity of the cytokines IL-11α and IL-1β [15]. Hence, all three conditions are marked by abnormal activation of the innate immune system resulting in severe inflammatory reactions. While DIRA most commonly presents in the neonatal period, DITRA is most common during childhood [15]. However, both conditions can present with disseminated pustular lesions, closely resembling GPP [15]. Nail changes have also been reported in both conditions, while bone changes and central nervous system manifestations are observed in DIRA alone [15]. Both conditions can also present with severe systemic symptoms, including fever and elevations in inflammatory markers [15].

The pathophysiology of AGEP has not been completely established but involves drug-specific CD4^+^ and CD8^+^ T-cells [20]. A variety of drugs have been implicated in the development of AGEP, but the most reported triggers include anti-infective agents (e.g., beta-lactams and macrolides), antimalarial drugs, and diltiazem [17]. Following their activation, drug-specific T-cells migrate to the skin, resulting in keratinocyte apoptosis and epidermal vesicle formation [20]. The vesicles are converted into sterile pustules as neutrophils are recruited to the area following massive release of interleukin (IL)-8 and granulocyte-macrophage colony-stimulating factor (GM-CSF) from CD4^+^ cells and keratinocytes [19]. The release of interferon-gamma (IFN-γ) from CD4^+^ cells also stimulates the secretion of IL-8 from nearby keratinocytes [19]. Th17 cells are also thought to play a role in the pathogenesis of AGEP. An increase in peripheral blood Th17 cells and their respective cytokine, IL-22, has been demonstrated in patients with AGEP [21]. Both IL-17 and IL-22 synergistically stimulate the production of IL-8 from keratinocytes and the recruitment and activation of neutrophils [21].

A small fraction of patients with AGEP have mutations in the IL36RN gene, which encodes the interleukin-36 receptor antagonist (IL-36Ra) [20]. In these patients, IL-36 signaling proceeds in an uncontrolled manner and leads to an increase in a variety of pro-inflammatory cytokines, including IL-6, IL-8, IL-1α, and IL-1β [20]. The increased production of these cytokines may predispose patients to the development of AGEP [20]. Genetic mutations in IL36RN have also been associated with GPP17, suggesting that AGEP and GPP share similar pathogenetic pathways reflected in their overlapping clinical phenotypes.

## 3. Clinical Features

Although each subtype of PP is united by the presence of sterile pustules composed of neutrophils, they present with unique clinical manifestations.

**Figure 1:**
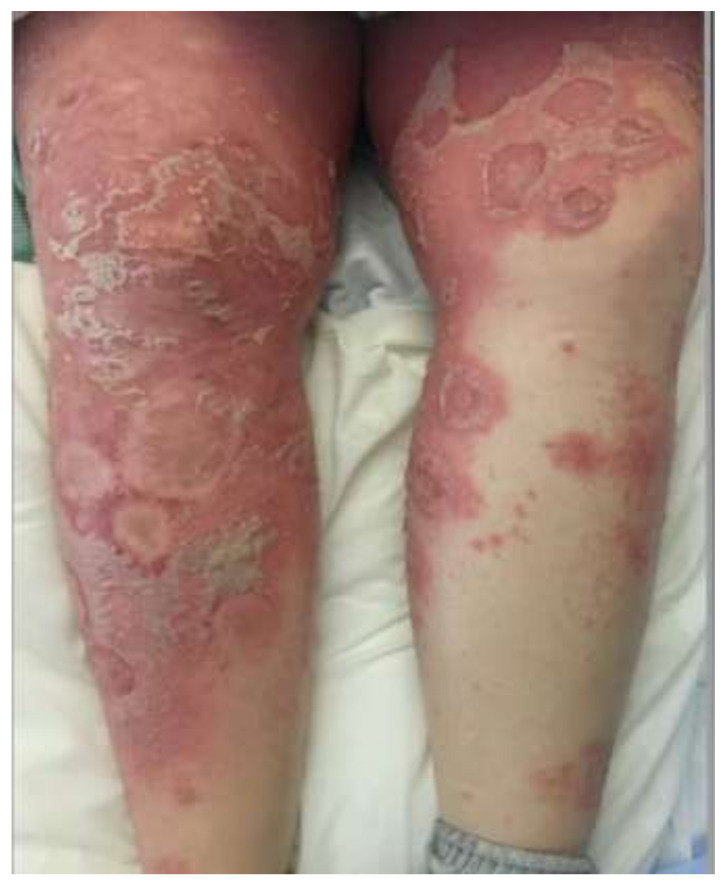
Generalized pustular psoriasis. Erythematous plaques covered by pustules, which demonstrate confluence.

GPP (Figure 1) is marked by abrupt flares of disseminated, painful cutaneous eruptions that spread rapidly [3,4]. The lesions are erythematous and covered with aseptic pustules that are distributed both along the edges and overlying the lesions [4,8]. GPP can present with severe systemic symptoms, including fever, malaise, fatigue, and arthritis [3,4]. Several weeks after the initial onset of pustulation, the pustules resolve with desquamation and collarettes of scales, finally resulting in hyperpigmentation [3,4,8]. Episodes tend to recur over the years, and lesions typically involve the entire body. Reported extracutaneous manifestations include cholestasis, cholangitis, epigastric pain, interstitial pneumonitis, oral erosions, acute renal failure, otitis media, uveitis, and osteoarthritis [3,4]. GPP can occur at any age, though it typically presents during the fifth decade of life with a slight female predominance [4]. GPP is the most severe form of PP, with a mortality rate of 7% in a recent retrospective review of 102 patients [28]. However, the authors note that their mortality rate is likely underestimated [28]. As such, they include morality data from several other clinical studies, with the reported mortality rates ranging from 2% to 16% [28]. Triggers for flares include infection, medications, stress, corticosteroid withdrawal, environmental agents, and pregnancy [4]. In pregnancy, GPP is referred to as IH [3,4]. IH most commonly occurs in the third trimester and can have life-threatening effects on both the fetus and the mother [3]. IUGR, miscarriage, and even fetal death have been reported, all likely due to the effects of placental insufficiency [3,4].

PPPP and ACH are both considered localized forms of PP that occur in adulthood [5]. PPPP is characterized by 1 to 10 mm pustules intermixed with yellow-brown macules on the palms and soles bilaterally [7,8,15]. In more severe cases, diffuse involvement of the palmoplantar surfaces is present [7,29]. Symptoms may include burning, pain, itching, bleeding, and superinfections [7]. The clinical course typically progresses through the development of sterile pustules on an erythematous base, followed by scaling, crusting, and fissuring [7]. The pustules often coalesce and resolve by forming yellow-brown macular lesions after several days [3,7]. The presence of nail psoriasis ranges from 30–76%, while psoriatic arthritis is seen in 8.6 to 26% of patients [7]. PPPP is associated with synovitis, acne, pustulosis, hyperostosis, osteitis (SAPHO), a rare condition involving the sternocostal joints and manubrium that is more commonly seen in Asian patients [7,29]. Strong links to smoking have been reported [3], with 79.8% of patients reporting a smoking history in a study by Twelves et al. [5]. Similar to GPP, there is also female predominance in both ACH and PPPP, although it is highest in PPPP compared to both GPP and ACH, with a ratio of 3.5:1 [4,5]. PPPP is a recurrent disease, and disease severity likely does not decrease over time [7]. ACH presents with pustular lesions overlying erythematous, scaling skin on the tips of fingers, and more rarely, the tips of toes [3,4,8]. Pustule formation has also been shown to occur beneath the nail plate in ACH, leading to nail bed destruction and the potential for bony erosions, necessitating early treatment to avoid permanent damage [3,8].

APP is marked by a relapsing course, although the overall prognosis is favorable [16]. APP presents most commonly in children, although it can affect individuals throughout adulthood as well [16]. In general, cutaneous eruptions tend to spread centrifugally, resolving after a few weeks [8,16]. Similar to the other forms of PP, pustules overlie erythematous areas of the skin, although they are predominantly located along the circumference of the lesions [16]. Most commonly, the lesions are found on the limbs, buttocks, and abdomen, with relapses occurring in areas adjacent to the initial lesions [16]. Similar to GPP, APP can present with systemic symptoms, such as fever and malaise, although they are far milder than those accompanying GPP [16].

**Figure 2:**
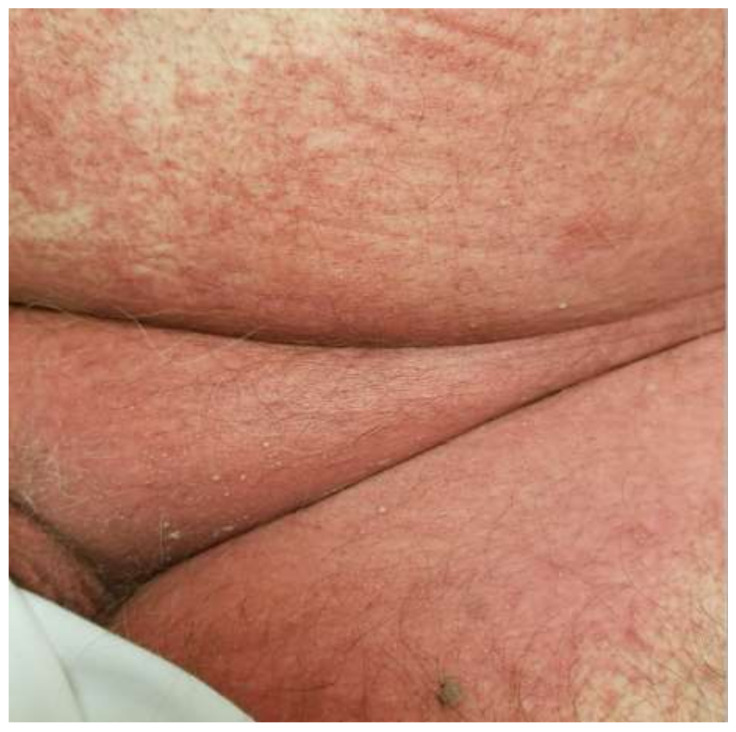
Acute generalized exanthematous pustulosis due to clindamycin. Early features include flexural erythema and small monomorphous pustules.

The clinical features of AGEP are outlined in the EuroSCAR criteria, which seeks to offer an algorithmic approach in the differentiation of AGEP from other SCARs and pustular eruptions. Classically, AGEP presents with many pinpoint non-follicular pustules scattered on an erythematous base, beginning in the flexural areas (Figure 2) [17]. The confluence of pustules can lead to large sheets of desquamation that mimic a positive Nikolsky sign [17]. Cases of atypical AGEP have been called TEN-like or DIHS-like AGEP overlap syndromes due to the heterogeneous presentation of AGEP [17]. Atypical features that can be seen include significant facial edema, purpuric lesions, target-like lesions, and extensive desquamation [18]. Mucous membrane involvement can be seen in 20% of cases but is almost always mild without significant sequelae [17]. 

The development of AGEP progresses rapidly, on a timeline of one to several days, although non-antibiotic cases of AGEP–particularly those cases due to hydroxychloroquine or other antimalarials-may have a significantly longer time to onset. Fever (>38 °C) and neutrophilia (>7 × 109 cells/l) are common laboratory findings. However, significant internal organ involvement is uncommon. Lymphadenopathy, mild hepatitis, and an increased creatinine clearance can be observed [18]. In a study of 58 patients with AGEP, 10 patients developed at least one systemic involvement (hepatic, renal, or pulmonary) [30]. 

Pustules spontaneously resolve within two weeks and are associated with superficial desquamation or collarettes of scale. According to EuroSCAR criteria, onset of rash > 10 days from initiation of the perpetrator drug, or a resolution of rash that takes > 15 days, favor a diagnosis other than AGEP. AGEP is generally self-limited, and resolution of lesions occurs within one to two weeks following drug withdrawal [19,30]. Mortality is less than 5% in AGEP with few lasting comorbidities [19].

## 4. Histopathologic Features

**Figure 3:**
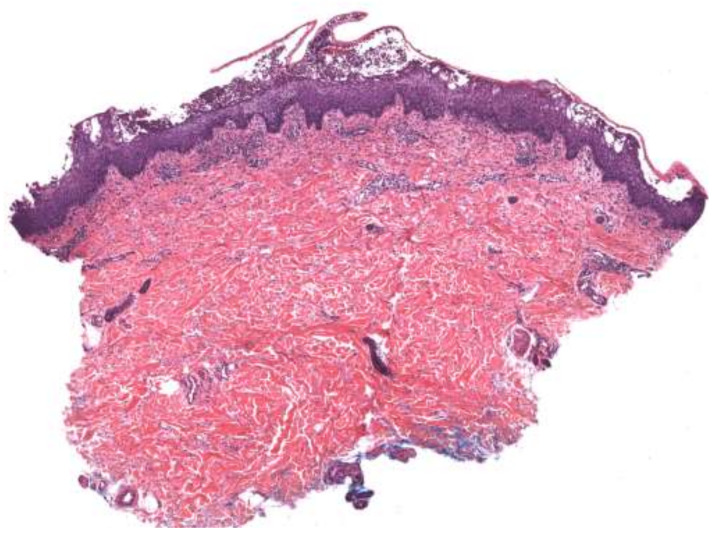
Generalized pustular psoriasis. Subcorneal pustular dermatitis with irregular epidermal hyperplasia and sparse dermal infiltrate.

On histopathology, PP (Figure 3) is characterized by neutrophilic infiltrates with the presence of spongiform pustules of Kogoj in the stratum spinosum and microabscesses of Munro with the stratum corneum [3,4], both of which constitute the hallmark features of active psoriasis [8]. The accumulation of neutrophils is readily observed in the stratum corneum, surrounded by parakeratosis [8]. APP specifically presents with subcorneal pustules, while PPPP additionally demonstrates eosinophilic and mast cell infiltrates in the upper dermis [4]. PP shows the same characteristic epidermal changes of PV, with psoriasiform elongation of the rete ridges and broad parakeratosis. However, instead of the smaller micropustules seen in PV, larger macropustules are seen in the variants of PP [31]. 

**Figure 4:**
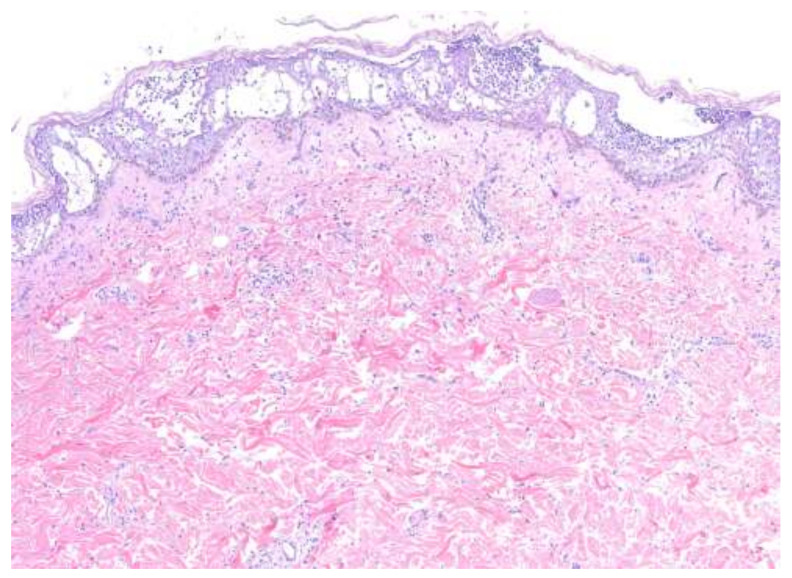
Acute generalized exanthematous pustulosis. Spongiform pustules and papillary dermal edema are consistent features. Orthokeratosis reflects the acuity of this process.

The histopathologic features of PP and AGEP share many similarities and, at times, may be indistinguishable. Biopsies of AGEP (Figure 4) characteristically show subcorneal and intraepidermal spongiform pustules according to EuroSCAR histopathologic criteria. In a study of 102 cases of AGEP, subcorneal pustules, intraepidermal pustules, or a combination of the two were seen in 41%, 20%, and 38% of cases, respectively [22]. Psoriasiform hyperplasia with rete ridge elongation and clubbing was seen in 76% and 51% of cases, respectively. Papillary dermal edema and a mixed inflammatory infiltrate are seen in the vast majority of cases. 

## 5. Differential Diagnosis

The differential diagnosis of AGEP presents a challenging clinical problem. Many of the histopathologic and clinical features of AGEP overlap with other pustular dermatologic conditions like PP, Sneddon-Wilkinson disease, IgA pemphigus, and bullous tinea. It is particularly important to distinguish AGEP from PP since these two conditions vary in their clinical course and treatment but share significant overlapping features [32]. A comparative study by Isom et al. examined the histopathologic and clinical features of 22 patients with AGEP and 11 patients with PP. Histopathologic findings that supported a diagnosis of AGEP included eosinophilic spongiosis, vacuolar interface dermatitis, and dermal eosinophilia [32]. The presence of at least 10 dermal CD161^+^ cells per punch biopsy specimen strongly favored a diagnosis of PP [32]. Clinically, a history of psoriasis and evidence of scaling plaques favored PP as a diagnosis, while mucosal involvement, although uncommon, was a feature unique to AGEP [32]. Of note, within the EuroSCAR study, 7% of patients had a personal history of psoriasis, indicating that patient history should not solely guide the differentiation between AGEP and PP [18].

Subcorneal pustular dermatosis (SPD) or Sneddon-Wilkinson disease is a rare but chronic pustular eruption that typically presents abruptly with flaccid pustules overlying normal-appearing skin [33]. SPD presents similarly to PP, with some arguing it is a variant of PP, particularly the annular variant with pustules spreading centrifugally [33]. In contrast to the pinpoint pustules seen in PP and AGEP, the hypopyon pustules of SPD contain clear fluid overlying purulent material at the base [33]. SPD cannot be reliably distinguished histologically from PP, but a lack of psoriasiform hyperplasia, mitotic figures, spongiform pustules, and telangiectasias favors SPD [33]. In addition, SPD lacks the presence of necrotic keratinocytes, vacuolar interface, and vasculitis that are variably identified in AGEP [33].

IgA pemphigus may only be differentiated from the classic form of SPD using immunofluorescence studies showing intercellular IgA deposition. In the SPD variant of IgA pemphigus, human desmocollin 1 is the autoantigen targeted by IgA autoantibodies [23]. Histopathologic examination typically shows subcorneal neutrophilic pustules, minimal acantholysis, and a mixed inflammatory infiltrate in the dermis [34].

Bullous tinea is an uncommon dermatophyte infection that typically presents with inflammatory subcorneal or intraepidermal vesicles and bullae on the soles [24]. Uncommonly, the clinical presentation of a bullous dermatophyte reaction can appear similar to a localized patch of AGEP or PP given the pustules on an erythematous base. The primary diagnostic test is a potassium hydroxide (KOH) preparation of the affected area, although fungal culture or skin biopsy may be considered if segmented hyphae are not detected, and clinical suspicion remains high.

In summary, differentiating PP and AGEP can present a significant diagnostic challenge. Both PP and AGEP present with widespread sterile pustules and may be accompanied by fever, malaise, and leukocytosis. Currently, there is no decisive feature that clearly distinguishes PP from AGEP, but a few clinical and histopathologic features can aid the clinician with discerning between these two entities. A history of psoriasis, the presence of scaling plaques, and the presence of dermal CD161^+^ cells favor a diagnosis of pustular psoriasis [32]. However, a diagnosis of AGEP is more likely if the histopathologic features include eosinophilic spongiosis, vacuolar interface dermatitis, and dermal eosinophilia [32].

## 6. Treatment

Traditionally, PP is treated with oral retinoids like acitretin and topical corticosteroids [4]. Additional agents that are used include cyclosporine, methotrexate, corticosteroids, TNF-alpha inhibitors, and more recently, IL-17 inhibitors [3,4]. Although PP typically requires systemic therapy, topical agents such as calcipotriene and corticosteroids are favored as first-line treatments in the localized forms of PP, most often ACH [3,4].

Recent advancements in the understanding of disease pathogenesis have led to new therapeutic approaches that can be tailored to specific mediators of the pathologic pathways. However, the lower prevalence of PP has limited the data on its treatment in comparison to the multitude of targeted biologic therapeutics for PV [3]. Specifically, anti-IL36 receptor monoclonal antibodies are currently under development for the treatment of PP [3,9]. In a study by Bachelez et al., these monoclonal antibodies showed promising results in seven patients with GPP. The patients were followed weekly, and the severity of their lesions was rated based on the Generalized Pustular Psoriasis Physician Global Assessment (GPPGA), with a score of 0 corresponding to clear skin. By week four, all seven patients received a score of 0. Of note, four of these seven patients did not have a mutation in the IL36RN gene, suggesting that IL-36 plays a role in PP pathogenesis regardless of the presence of mutational status [9]. 

Traditional biologic therapy for PV can also be applied to PP. For example, adalimumab, a TNF-alpha inhibitor, has shown efficacy in the treatment of GPP and PPPP in small numbers of patients [35,36,37,38]. Similarly, ustekinumab, an anti-IL-12/23 monoclonal antibody, has shown efficacy in treating recalcitrant GPP [39]. Anti-psoriatic drugs targeting the IL-17 pathway (secukinumab, ixekizumab, and brodalumab) have all shown positive results as potential alternative treatments for PP, although data remains limited with most studies restricted to the Japanese population [40,41,42]. For example, an open-label study showed that 10 out of 12 adults with GPP showed clinical global impression ratings of “much improved” or “very much improved” at week 16, with the initiation of secukinumab [40]. Interestingly, there are case reports of paradoxical flarings of PP attributed to the use of TNF-alpha, IL-12/23, and IL-17 inhibitors [43,44,45], necessitating cessation of the implicated drug and initiation of alternative treatments like cyclosporine. 

The newest biologic treatments developed for PV target IL-23, including guselkumab, risankizumab, and tildrakizumab. Similar to other biologics, their use has been extrapolated to the treatment of PP. For example, guselkumab has demonstrated promising results for patients with PPPP and GPP [7]. Guselkumab was shown to be efficacious in a phase III RCT in Japanese patients with PPPP [7]. The Palmoplantar Psoriasis Area and Severity Index (PPPASI) was used to evaluate patient response to guselkumab and 52. Fifty-seven percent of patients saw at least a 50% improvement in total PPPASI score (PPPASI-50) at week 16 [7]. Ten Japanese patients with GPP were enrolled in a phase III, open-label study that evaluated the efficacy and safety of guselkumab [11]. At week 16, seven of the nine remaining patients had achieved treatment success, defined as a Clinical Global Impression score of “very much improved”, “much improved”, or “minimally improved” [11].

Other potentially therapeutic targets include IL-1β and the IL-1 receptor. Two monoclonal antibodies have been designed to bind to IL-1β: gevokizumab and canakinumab. Two patients with severe GPP who received gevokizumab as part of an open-label, expanded-access study “had a respective 79% and 65% reduction in GPP area and severity index scores at weeks 4 and 12” [12]. Canakinumab therapy has been reported to significantly improve the lesions of a patient with GPP who previously failed therapy with anakinra [13]. Anakinra is an IL-1 receptor antagonist that may prove to be efficacious for the treatment of GPP and ACH, although its efficacy and safety remain to be evaluated with further RCTs [13].

Apremilast is a small-molecule inhibitor of phosphodiesterase (PDE) 4 that is currently approved for the treatment of plaque psoriasis and psoriatic arthritis. There are limited case reports that demonstrate the therapeutic potential of apremilast for patients with PPPP and GPP [46,47,48]. APLANTUS was a 20-week, phase II open-label study that investigated the efficacy and safety of apremilast in patients with PPPP [14]. Patients who were enrolled saw a significant decrease in PPPASI, with 61.9% of patients achieving PPPASI-50 [14].

The most important initial step in treating AGEP is the removal of the offending agent [20,25]. Upon drug cessation, symptoms typically begin to improve within a few days [20,25]. AGEP is generally self-limiting with a favorable prognosis, although it may be severe enough to require hospitalization [49]. In these cases, supportive care, specifically infection prevention, is crucial when pustules begin to coalesce and larges sheets of skin desquamate. During the pustular phase, lesions should be covered with moist, antiseptic dressings [20,50]. Antibiotics should only be implemented if superinfection of the pustules is suspected. In patients with pruritis and inflammation, topical corticosteroids are useful [20]. In a large retrospective study, authors demonstrate the use of potent topical steroids associated with a decrease in median hospital duration [51]. In severe or refractory cases, systemic corticosteroids or cyclosporine are useful in accelerating disease clearance [25,50].

## Figures and Tables

**Table 1:** Features of pustular psoriasis variants and acute generalized exanthematous pustulosis.

Disease	Clinical Morphology	Demographic	Pathology	Treatment
Generalized pustular psoriasis (GPP)	All PP subtypes contain sterile pustules [3]. Disseminated, painful erythematous lesions covered with aseptic pustules [3,8]. Severe systemic symptoms, including fever, malaise, fatigue, and arthritis, may be present [3,4].	Fifth decade of life with slight female predominance [4].	Pathogenesis: Disruption of the interleukin-36 pathway plays a major role (mutations in IL36RN), although there is significant heterogeneity in the gene pathways implicated [3,9,10]. The innate immune system, environmental factors, and genetic susceptibility all contribute [7]. Histopathology: Spongiform pustules of Kogoj in the epidermis and microabscesses of Munro [3,4]. Parakeratosis and psoriasiform hyperplasia [8].	Topical corticosteroids, oral retinoids (i.e., acitretin), cyclosporine, methotrexate, TNF-α inhibitors (i.e., adalimumab), anti-IL-17 monoclonal antibody (i.e., secukinumab), anti-IL-23 monoclonal antibody (i.e., guselkumab), anti-IL-1β monoclonal antibodies (i.e., gevokizumab and canakinumab), IL-1R inhibitor (i.e., anakinra), PDE-4 inhibitor (i.e., apremilast) [3,4,7,9,11,12,13,14].
Impetigo herpetiformis	All PP subtypes contain sterile pustules [3]. See GPP.	GPP during third trimester of pregnancy [3].	Pathogenesis: see GPP. Histopathology: see GPP.	Cyclosporine, systemic corticosteroids [3,4]
Palmoplantar pustular psoriasis (PPPP)	All PP subtypes contain sterile pustules [3]. Pustules intermixed with yellow-brown macules on palms and soles [7,8,15].	Slight female predominance [4,5]	Pathogenesis: Mutations in IL36RN make up a significantly smaller proportion of cases compared to GPP [4,5]. Mutations in AP1S3 and CARD14, as well as abnormalities of eccrine sweat glands, have been implicated in PPPP [7]. Histopathology: see GPP; on acral skin.	See GPP.
Acrodermatitis continua of Hallopeau	All PP subtypes contain sterile pustules [3]. Pustular lesions overlying erythematous, scaling skin on the tips of the fingers and toes [3,4,8].	Slight female predominance [4,5].	Pathogenesis: see GPP. Histopathology: see GPP, on acral skin.	Topical corticosteroids, calcipotriene [3,4]
Annular pustular psoriasis	All PP subtypes contain sterile pustules [3]. Pustules located circumferentially on erythematous skin lesions. Lesions present on limbs, buttocks, abdomen. Can present with fever and malaise [16].	More common in children [16].	Pathogenesis: see GPP. Histopathology: see GPP.	See GPP.
Acute generalized exanthematous pustulosis	Sterile, pin-sized pustules overlying edematous and erythematous skin. Often appears on the face or intertriginous areas before spreading to the trunk and limbs [17]. Acutely accompanied by fever, neutrophilia, and eosinophilia [18].	More common in adults with a slight female predominance [17].	Pathogenesis: Drug-specific T-cell predominantly infiltrates with neutrophil accumulation mediated by IL-8 and GM-CSF [19,20,21]. Th17 cells are also involved in neutrophil activation [21]. Mutations in *IL36RN* found in some patients [20]. Histopathology: Spongiform subcorneal or intraepidermal pustules ± necrotic keratinocytes, vacuolar interface dermatitis, dermal eosinophilia, psoriasiform hyperplasia [17,22].	Typically resolves within 2 weeks of discontinuation of the offending drug [20,23]. Topical steroids are often used for symptomatic relief [20]. Systemic corticosteroids or cyclosporine are useful in severe cases or with extracutaneous involvement [24,25].

GPP: generalized pustular psoriasis; PP: pustular psoriasis; PPPP: palmoplantar pustular psoriasis.
